# Patterned Feeding of a Hyper-Palatable Food (Oreo Cookies) Reduces Alcohol Drinking in Rats

**DOI:** 10.3389/fnbeh.2021.725856

**Published:** 2021-10-20

**Authors:** Zoela Leon, Krishna Shah, Lauren S. Bailey, Anushree N. Karkhanis, Sunil Sirohi

**Affiliations:** ^1^Laboratory of Endocrine and Neuropsychiatric Disorders, Division of Basic Pharmaceutical Sciences, College of Pharmacy, Xavier University of Louisiana, New Orleans, LA, United States; ^2^Department of Psychology, Behavioral Neuroscience Program, Center for Developmental and Behavioral Neuroscience, Binghamton University—SUNY Binghamton, Binghamton, NY, United States

**Keywords:** alcohol use disorder, high-sugar/fat diet, palatable diet, alcohol drinking, dopamine, nucleus accumbens

## Abstract

While a bidirectional positive link between palatable food intake and alcohol drinking has been suggested, several rodents studies report reduced alcohol drinking following palatable diets exposure. These studies utilized purified rodents’ diets high in sugar/fat; however, the effects of hyper-palatable food (HPF) rich in fat and sugar on alcohol drinking remain unclear. Furthermore, neural substrates involved in HPF-mediated changes in alcohol consumption are poorly understood. Therefore, the present study evaluated the effects of patterned feeding of a hyper-palatable food (Oreo cookies) on alcohol drinking as well as dopamine (DA) and serotonin (5-HT) content in rat’s mesocorticolimbic (medial-prefrontal cortex, orbitofrontal cortex, amygdala, and nucleus accumbens) circuitry. Male Long Evans rats received 8-weeks of intermittent (Mon, Tue, Wed) Oreo cookies access, which induced a patterned feeding, in which rats in the Oreo group overconsumed calories on HPF days whereas underconsumption was observed on chow only (Thu, Fri) days. Following HPF exposure, alcohol consumption was evaluated while patterned feeding continued. Alcohol intake in the Oreo group was significantly lower as compared to the chow controls. However, alcohol intake in the Oreo group increased to the levels seen in the group receiving chow following the suspension of patterned HPF feeding. Finally, DA levels in the nucleus accumbens were significantly greater, whereas its metabolite (DOPAC) levels were lower in the Oreo group compared to the chow controls. Surprisingly, 5-HT levels remained unaltered in all tested brain areas. Together, these data suggest that HPF-associated increased DA availability and reduced DA turnover within mesocorticolimbic circuitry may regulate alcohol drinking following patterned HPF feeding.

## Introduction

Problematic caloric intake is not only a core component of some eating disorders but also is linked to several public health concerns. For example, binge eating disorder, characterized by consuming a large amount of food in a short period with a behavioral loss of control over eating, can significantly impact overall health, quality of life, and healthcare costs (Ágh et al., 2016). Hyper-palatable foods (HPF), rich in sugar and fat, are the typically preferred foods consumed during these episodes (Leigh et al., 2018) and data suggest that individuals who engage in such problematic eating behavior are at higher risk for developing substance abuse, overweight/obesity, and worsening depressive symptoms (Ross and Ivis, 1999; Swanson et al., 2011; Skinner et al., 2012; Mehlig et al., 2018). Furthermore, substance use disorder frequently co-occurs with eating disorders (Bulik et al., 2004; Harrop and Marlatt, 2010), and a significant proportion of the college-aged population has been reported to engage in episodes of dysregulated drinking/eating, an experience that could trigger alcohol/drug abuse and numerous health concerns (Callas et al., 2004; Ferriter and Ray, 2011; Kelly-Weeder, 2011).

Converging research evidence suggests common neurochemical, behavioral, and physiological determinants of maladaptive eating and alcohol drinking (Fortuna, 2010; Morganstern et al., 2011; Nogueiras et al., 2012). For instance, feeding peptides, traditionally known for controlling appetite and energy metabolism, also regulate the intake and reinforcing properties of alcohol (Vadnie et al., 2014). It is also becoming apparent that hyper-palatable foods can interact with brain reward circuitry (Volkow et al., 2012), and changes in several of these neurochemical systems have been reported following HPF and alcohol intake (Barson et al., 2011; Volkow et al., 2012, 2017). For example, both food and drug reward stimulate DA release in the nucleus accumbens (NAc; Hernandez and Hoebel, 1988; Yoshimoto et al., 1992; Rada et al., 2005; Liang et al., 2006), and alterations in DA turnover and DA receptors gene expression have been reported following prolonged alcohol and HPF exposure (Hajnal and Norgren, 2002; Vasconcelos et al., 2003; Davis et al., 2008; Villavasso et al., 2019). Similarly, serotonergic (5-HT) neurotransmission is also implicated in mediating behavioral and emotional impairments following chronic exposure to alcohol and high-calorie diets (Kurhe and Mahesh, 2015; Zemdegs et al., 2016; Li et al., 2020; Popova et al., 2020). Together, these studies suggest that both hypercaloric foods and alcohol may affect central monoaminergic systems, and neuroadaptations in the mesocorticolimbic circuitry could mediate the effects of chronic HPF and alcohol exposure.

Impaired emotional status resulting from chronic alcohol exposure has been shown to promote escalated alcohol intake (Kissler et al., 2014). Considering that prolonged dysregulated eating of HPFs is capable of triggering negative emotional states (Cottone et al., 2009; Iemolo et al., 2012), excessive consumption of hyper-palatable food and resultant changes in neuroendocrine signaling along with negative affective states could trigger alcohol use disorder (AUD). Interestingly, some studies have suggested a bidirectional positive link between HPF intake and alcohol drinking (Pekkanen et al., 1978; Mitchell et al., 1985; Krahn and Gosnell, 1991; Avena et al., 2004; Carrillo et al., 2004). However, studies have also demonstrated reduced alcohol drinking following both sugar- and fat-rich palatable diets exposure (Yung et al., 1983; DiBattista and Joachim, 1999; Stickel et al., 2016; Takase et al., 2016; Cook et al., 2017; Gelineau et al., 2017; Sirohi et al., 2017b; Villavasso et al., 2019; Shah et al., 2020). It is important to note that several procedural/experimental differences among these studies could explain the differential impact of palatable diets on alcohol drinking and have been reviewed recently (Brutman et al., 2020). Briefly, some studies reported increased alcohol intake provided palatable diets in the acute or chronic manner and assessed alcohol drinking following palatable diets suspension (Pekkanen et al., 1978; Carrillo et al., 2004). On the other hand, studies from our lab provided intermittent access and evaluated alcohol drinking while rats were still maintained on intermittent palatable diet cycling (Sirohi et al., 2017b; Villavasso et al., 2019). Furthermore, hedonic fat or sugar consumption has been suggested to produce fundamentally different behavioral states (Avena et al., 2009). In this regard, a decrease in anxiety-like behavior has been reported by studies providing intermittent access to the high-fat diet in a non-consecutive manner (Mon, Wed, Fri; Sirohi et al., 2017b), whereas when a high-sugar diet was provided in an intermittent but on two consecutive days in a week, increase in anxiogenic behavior was reported (Cottone et al., 2009). In short, how hyper-palatable food overconsumption impact behavioral processes that regulate alcohol drinking is poorly understood.

While most of these studies provided rodent’s purified diets high in sugar or fat, typical dysregulated feeding episodes in the real-world involve hyper-palatable food rich in both fat and sweet. Therefore, the present study evaluated patterned feeding of real-world hyper-palatable food (Oreo double stuffed cookies) on alcohol drinking and monoamines (DA and 5-HT) levels in rat’s corticolimbic areas. Previously, we have shown that a 2-week of patterned high-fat diet pre-exposure is sufficient to reduce alcohol drinking in rats (Villavasso et al., 2019). Considering that the length and exposure history of a calorie-rich food can produce fundamentally different behavioral outcomes (Tracy et al., 2015; Krishna et al., 2016), alcohol drinking in the present study was evaluated following extended (three times a week for 10-weeks), patterned HPF administration. It was hypothesized that patterned feeding of hyper-palatable food would increase alcohol drinking by associated changes in the monoamines content in the brain reward circuitry.

## Materials and Methods

### Animals

Male Long Evans rats (Envigo RMS, Inc, Indianapolis, IN) initially weighing ~300 g were used. Animals were individually housed in a temperature (~70°F) and humidity (~60%) controlled vivarium on a standard 12 h reverse light-dark cycle (lights on at 1:00 AM and off at 1:00 PM). Upon arrival, animals were gently handled before any experimental manipulation, and baseline body weight, food intake, and water intakes were recorded. Cage cleaning and changes occurred every Monday ~10:30 AM. All procedures were approved by the Institutional Animal Care and Use Committee guidelines at the Xavier University of Louisiana.

### Diet

All animals received *ad libitum* access to standard rodent chow (Tekland-Envigo Diets #2020X, 3.1 kcal/g with 16% Calories from fat and 60% Calories from carbohydrates) and water. Rats in the experimental group also received intermittent access to Double Stuff Oreo cookies (Walmart, 4.83 kcal/g with 24% Calories from fat and 72% Calories from carbohydrates, of which 45% Calories were derived from sugar). Other test diets used in the study were high-fat diet (HFD; Research Diets #D03082706, 4.5 kcal/g with 40% Calories from fat and 46% Calories from carbohydrates, of which 7.9% Calories were derived from sugar) and high-sugar diet (HSD; Research Diets #D10001, 3.9 kcal/g with 11.5% Calories from fat and 67.7% Calories from carbohydrates, of which 51% Calories were derived from sugar).

### General Experimental Procedure

**Figure 1:**
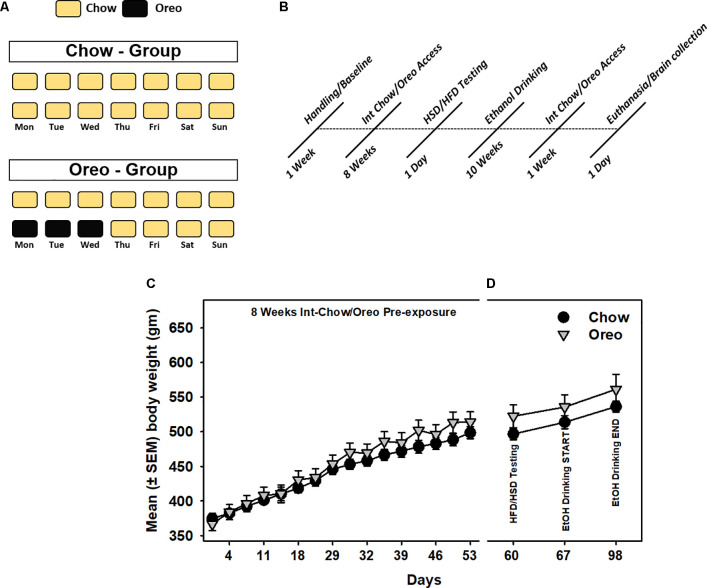
Schematic representation of patterned feeding paradigm and associated body-weight changes. **(A)** During the feeding paradigm, experimental group of rats (*n* = 5/group) received 24 h intermittent access (Mon, Tue, and Wed) to Double Stuff Oreo cookies whereas control group of rats received chow. **(B)** A timeline showing a series of behavioral and biochemical testing following initial 8 weeks of patterned feeding. All rats had *ad libitum* access to chow and water. Mean (± SEM) body weight readings during **(C)** initial 8 weeks of pre-exposure and **(D)** subsequent testing sessions. There was no significant difference between body weights of the control and the experimental group at any time point.

Male Long Evans rats (*n* = 5/group) with no significant between-group difference in body weight, food intake, and water intake were randomly divided into control (Chow) and hyper-palatable food (HPF/Oreo) groups. All rats had *ad libitum* access to standard rodent chow and water for the entire duration of the experiment. Rats in the HPF group also received intermittent (Mon, Tues, and Wed) access to Double Stuff Oreos cookies (3.0 cookies/session) ~2.0 h into the dark cycle. The control group received chow during this period. Standard chow was available to all rats for the rest of the week, as shown in Figure 1A. Food intake was manually recorded every 24 h, and body weight was measured every Monday and Thursday. Any leftover cookie crumbs were recovered from the cage at the end of HPF access. Following an initial 8 weeks of patterned HPF feeding, a series of tests (Figure 1B), as described below, were conducted while the animals still maintained on a patterned feeding schedule, unless noted otherwise. Rats were weighed simultaneously just before respective food/fluid presentation.

We first examined the impact of withdrawal from patterned feeding of an HPF exposure on high fat or high-sugar diet preference. In the 9th week, ~24 h following termination of HPF patterned feeding cycle, both control and the experimental groups received eight pellets of HFD and HSD (11:00 AM), and hourly food intake was recorded for 4 h. HFD and HSD testing occurred over a period around light dark switch so that food intake could be captured at least 2 h before and 2 h after this switch. These time points were chosen as rodents typically consume their biggest meals around this switch. Also, since the goal of the manuscript was to assess the effects of withdrawal from hyper-palatable food, 2 h post switch testing time point was chosen as it was ~24 time period following Oreo cookies access was suspended. All rats resumed patterned HPF cycling following this testing.

In the 10th week, alcohol drinking was evaluated on chow-only days (Thu, Fri, Sat) in a two-bottle choice paradigm, as explained below. Alcohol (20% v/v) and water bottles were provided in the rat’s home cages (~2.0 h into the dark cycle), and alcohol intake was manually recorded 2 or 24 h following administration. Alcohol testing was conducted for 5 weeks on chow-only days (Thu, Fri, and Sat) while patterned HPF cycling continued. Next, we examined alcohol drinking once dietary manipulations were released. For this, patterned HPF cycling was suspended (Mon, Tue, and Wed), and all animals received alcohol in a similar manner on regular chow only days. Following the conclusion of alcohol drinking studies, intermittent HPF cycling was reinitiated until animals were euthanized.

Finally, all animals were euthanized on chow access day (~24 h after the last patterned HPF cycle), and their brains were immediately snap-frozen. The medial prefrontal cortex (mPFC), orbitofrontal cortex (OFC), nucleus accumbens (NAc), and amygdala (Amyg) were micro- dissected and prepped for dopamine (DA), serotonin (5-HT), and their metabolites content analysis.

### Alcohol Drinking Procedure

Alcohol testing occurred using a two-bottle choice paradigm, as done previously (Sirohi et al., 2017b; Villavasso et al., 2019). On alcohol testing days, rats were given 20% v/v unsweetened alcohol and water bottles. The position of alcohol and water bottles was switched in each session to reduce and minimize conditioning effects on alcohol intake. Body weight and weight of alcohol and water bottles were manually recorded on testing days. Data represents 15 separate alcohol drinking sessions where 2 or 24 h alcohol intake was measured. Alcohol preference was calculated as alcohol intake/water intake.

### Brain Neurotransmitters Analysis

Brain tissue samples (*n* = 3–5/brain region) from the mPFC, OFC, NAc, and Amyg were collected using tissue punches and were weighed for wet weight. Tissue samples were then homogenized in 0.2 M Perchloric acid (1 ml EDTA, 10 ml 60% HClO_4_, filled to 500 ml with ultrapure water) using sonication. Homogenized samples were centrifuged for 15 min at 12,000 rpm and 4°C (Sorvall Legend Micro 21R Centrifuge; Thermo Scientific; Waltham MA). The supernatant was collected for analysis and the pellet was discarded. Samples were analyzed using high-performance liquid chromatography (HPLC). The mobile phase consisted of the following composition: 8% acetonitrile, 100 mM phosphoric acid, 100 mM citric acid, 0.1 mM EDTA.Na2, 600 mg/L octanesulfonic acid sodium salt, *pH* = 3.0. All chemicals used were HPLC grade and dissolved in 18.0 MΩ purity water. A sample volume of 5 μl sample was injected for analyses using an autosampler (AS 110, Antec, Zoeterwoude, Netherlands). Standards of known concentrations of dopamine and, 3,4-Dihydroxyphenylacetic acid (DOPAC), homovanillic acid (HVA), serotonin, and 5-HIAA were used to quantify concentrations of the respective monoamines and their metabolites in the brain samples. An ALEXYS Neurotransmitter Analyzer (Antec, Zoeterwoude, the Netherlands) consisting of a stationary phase C18 column (Acquity UPLC BEH, 1.7 μm diameter, and 1 × 100 mm length) and a DECADE Elite electrochemical detector (senCell 2 mm GC sb, Antec, Zoeterwoude, Netherlands). Clarity software (Prague, The Czech Republic) was used for quantifying neurotransmitter concentrations. Data are expressed as nM/mg of tissue weight.

### Statistical Analysis

A mixed-model two-way ANOVA analyzed body weight, food intake, and alcohol drinking data, with appropriate *post hoc* (Sidak) analysis. The within subject variable was time intervals, and the between-group variable was diet exposure. HFD/HSD testing data were analyzed by the two-way repeated measure of ANOVA, followed by *post hoc* analysis (Turkey). A Student’s *t*-test was also used wherever applicable, particularly for brain monoamine data analysis. Statistical comparisons were conducted at 0.05 α level using GraphPad Prism 7.05 (GraphPad Software Inc).

## Results

### HPF Patterned Feeding: Body Weight

A mixed-model two-way ANOVA identified a main effect of time (*F*_15,120_ = 575.1, *p* < 0.0001) and a significant HPF exposure × time interaction (*F*_15,120_ = 5.79, *p* < 0.0001) during 8 weeks initial intermittent HPF exposure compared to the chow controls. However, no significant (*p* > 0.05) between-group body weight differences existed during this period (Figure 1C). Similarly, no significant (*p* > 0.05) between group body weight differences existed during any of the testing time points (Figure 1D).

### HPF Patterned Feeding: Food Intake

**Figure 2:**
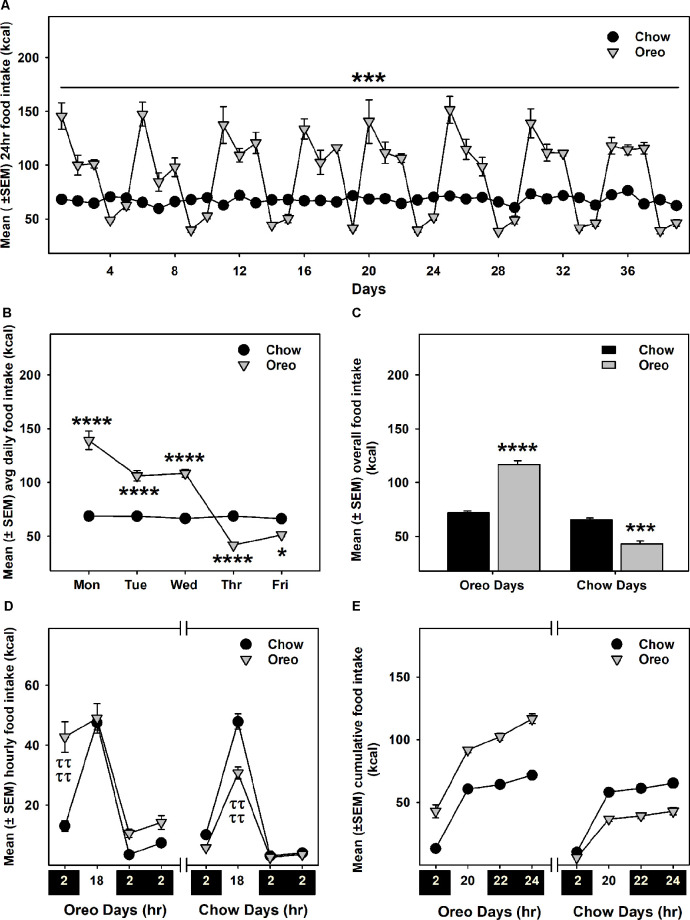
Energy intake during the 8-weeks of patterned HPF feeding. Data compare mean (± SEM) 24 h food intake (kcal) between chow and HPF group. **(A)** Rats in the HPF group followed a pattern where they significantly overconsumed on HPF access days (i.e., Mon, Tue, and Wed) and under-consumed on chow-only access days. ***p < 0.001 main effect of diet exposure. HPF group of rats significantly overconsumed and under-consumed on Oreo-access days and chow-access days respectively when data were compared as indicated by the average of daily food intake **(B)** and overall food intake during the exposure period **(C)**. *p < 0.05, ***p < 0.001, ****p < 0.0001 compared to chow controls. Food intake (kcal) was also measured at different timepoints after initiation and suspension of HPF respectively. Data represents (± SEM) hourly **(D)** and cumulative **(E)** food intake at these time-points. HPF rats significantly overconsumed during the first 2 h (0–2 h) on the Oreo days, and significantly under-consumed during the 2–18 h timeframe on Chow days. ^ττττ^*p* < 0.0001 compared to chow controls. ***p < 0.001, ****p < 0.0001 main effect of diet exposure. HPF, hyper-palatable food.

Intermittent HPF exposure induced a patterned feeding cycle, in which rats in the Oreo group overconsumed on HPF access days, whereas underconsumption was observed on chow only days (Figures 2A–C). A mixed-model two-way ANOVA identified a main effect of time (*F*_38,304_ = 25.11, *p* < 0.0001), a significant HPF exposure × time interaction (*F*_38,304_ = 24.01, *p* < 0.0001) and a significant between-group effect (*F*_1,8_ = 42.49, *p* < 0.001). On the other hand, the caloric intake of rats in the chow group was consistently similar during this period. Food intake was also measured on two separate weekends only and no significant between-group difference existed these days. Furthermore, significant (*p* < 0.0001) caloric overconsumption (~93% of kcal from Oreo cookies) occurred within 0–2 h of the presentation, whereas a significant (*p* < 0.0001) caloric underconsumption occurred 2–18 h following the suspension of HPF (Figures 2D,E). The caloric intake at other time points was identical in both groups.

### HPF Patterned Feeding: HFD/HSD Preference

**Figure 3:**
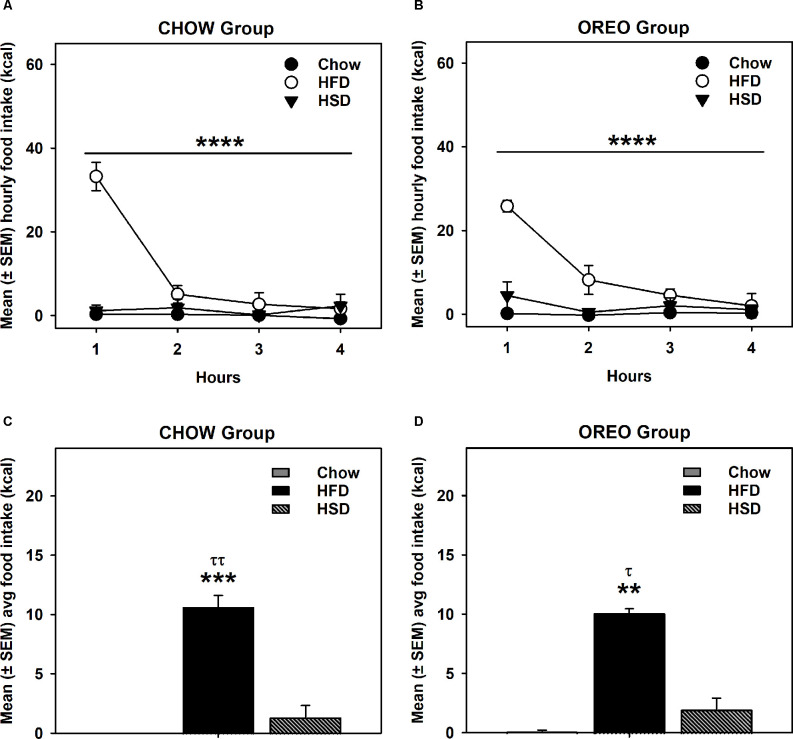
HFD/HSD preference during acute withdrawal from patterned HPF feeding. In the 9th week, 24 h after the termination of intermittent HPF access, rats were tested for their HFD/HSD preference. Data represent mean (± SEM) hourly and average food intake from each diet (chow, HFD, and HSD). A significant between-diet effect was noted in the hourly food intake in both Chow **(A)** and Oreo **(B)** groups. ****p < 0.0001 between diet effect. A significant preference of HFD over both HSD and chow was noted in both Chow **(C)** and Oreo **(D)** groups. However, no significant between diet type differences in HFD or HSD intake were evident. There was no significant preference for HSD compared to chow in either group. **p < 0.01, ***p < 0.001 compared to chow. ^τ^*p* < 0.05, ^ττ^*p* < 0.01 compared to HSD. HFD, high-fat diet; HSD, high-sugar diet.

HFD/HSD preference data were analyzed by a two-way repeated measure ANOVA. In the rats receiving chow only, there was a main effect of time (*F*_3,12_ = 20.98, *p* < 0.0001), a significant diet × time interaction (*F*_6,24_ = 20.51, *p* < 0.0001) and a significant between-diets effect (*F*_2,8_ = 73.9, *p* < 0.0001; Figure 3A). Similar results were obtained in the rats receiving intermittent access to Oreo, where there was a main effect of time (*F*_3,12_ = 15.07, *p* < 0.001), a significant diet × time interaction (*F*_6,24_ = 7.679, *p* < 0.001) and a significant between-diets effect (*F*_2,8_ = 98.2, *p* < 0.0001; Figure 3B). While the HFD intake in both Chow (*p* < 0.0001) and Oreo (*p* < 0.01) group of rats was significantly elevated compared to the chow and HSD intake, HSD intake was not significantly different compared to chow intake in either group (Figures 3C,D). Furthermore, HFD/HSD intake was not significantly (*p* > 0.05) different between the Chow and Oreo group of rats. These data suggest that while both the Chow and the Oreo group of rats preferred HFD over chow and HSD, this preference was not impacted by 8 weeks patterned HPF feeding.

### HPF Patterned Feeding: Alcohol Drinking

**Figure 4:**
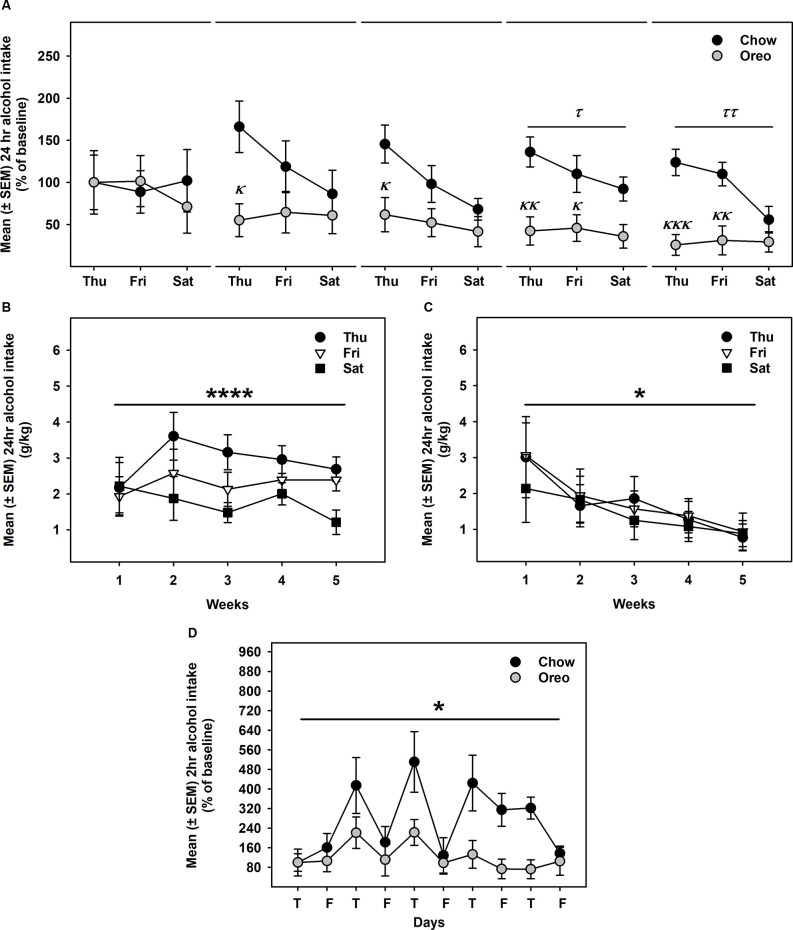
Alcohol drinking following patterned feeding of HPF. Alcohol (20% v/v) was provided, and intake was recorded on chow-only days (Thr, Fri, Sat) for 5 weeks. **(A)** Mean (±SEM) % of baseline (day 1) alcohol intake is plotted. While there was no significant difference in alcohol intake between groups in the first week, alcohol drinking was gradually reduced in the case of the Oreo group. During the 4th and the 5th week/session, the difference markedly increased with HPF rats significantly consuming less alcohol than the chow controls. ^τ^*p* = 0.01, ^ττ^*p* = 0.0068 main effect of diet. ^κ^*p* < 0.05, ^κκ^*p* < 0.01, ^κκκ^*p* < 0.001 compared to chow controls. Furthermore, alcohol consumption on each testing day was compared across the 5 weeks. **(B)** In chow group, a significant difference in alcohol consumption was noted among testing days, a decreasing order from Thursday to Saturday. No significant week’s effect was noted. ****p < 0.0001 main effects of testing days. **(C)** In the Oreo group, there was no difference in alcohol consumption over different testing days; however, there was a significant decrease in alcohol intake across the weekly testing sessions. *p < 0.05 effect of the week. **(D)** Similar to 24-h alcohol intake, 2-h alcohol intake in the Oreo group was significantly lower as compared to the chow controls. *p < 0.05 main effect of diet.

Alcohol drinking data were analyzed by a mixed-model two-way ANOVA, which identified a main effect of time (*F*_14,112_ = 2.74, *p* < 0.01), a significant diet × time interaction (*F*_14,112_ = 2.628, *p* < 0.01) and a non-significant trend between-group effect (*F*_1,8_ = 4.774, *p* = 0.06; Figure 4A). While alcohol drinking was not significantly different between groups in the first week, further analysis revealed a gradual reduction in alcohol drinking behavior over 5 weeks of testing. A significant between-group difference in alcohol drinking was observed on fourth (*F*_1,8_= 10.01, *p* = 0.01, power = 0.79) and fifth (*F*_1,8_= 13.12, *p* = 0.0068, power = 0.89) alcohol testing week. Interestingly, rats in the chow group repeatedly displayed escalated alcohol intake on renewed alcohol access (on Thursday), an effect reduced over the next testing days each week. To examine if alcohol drinking differed on testing days, we compared each alcohol drinking day (Thu, Fri, and Sat) across 5 weeks of alcohol testing in both chow and Oreo groups. In the chow group, a repeated measure two-way ANOVA identified a main effect of testing days (*F*_2,8_ = 40.36, *p* < 0.05) but no significant week’s effect (Figure 4B). Post-hoc analysis further revealed that alcohol intake on Thursday was significantly elevated compared to Friday (*p* < 0.01) and Saturday (*p* < 0.0001). In addition, Friday alcohol drinking was also elevated compared to Saturday (*p* < 0.05). On the other hand, in the case of Oreo group, a repeated measure two-way ANOVA identified a main effect of the week (*F*_4,16_ = 3.671, *p* < 0.05) but no significant testing day effect (Figure 4C). *Post hoc* analysis further revealed that alcohol intake in the 4th (*p* < 0.05) and the 5th (*p* < 0.01) week was significantly reduced compared to the 1st week in the Oreo group. Two-hour alcohol intake was also evaluated on Thu and Fri and a mixed-model two-way ANOVA identified the main effect of time (*F*_9,72_ = 5.458, *p* < 0.0001), a significant food × time interaction (*F*_9,72_ = 2.163, *p* < 0.05), and a significant between-group effect (*F*_1,8_ = 6.414, *p* < 0.05; Figure 4D). Similar to 24 h alcohol intake data, alcohol drinking in the chow group significantly escalated on renewed alcohol access, an effect absent in the Oreo group of rats. Total fluid intake was also not significantly different between groups.

**Figure 5:**
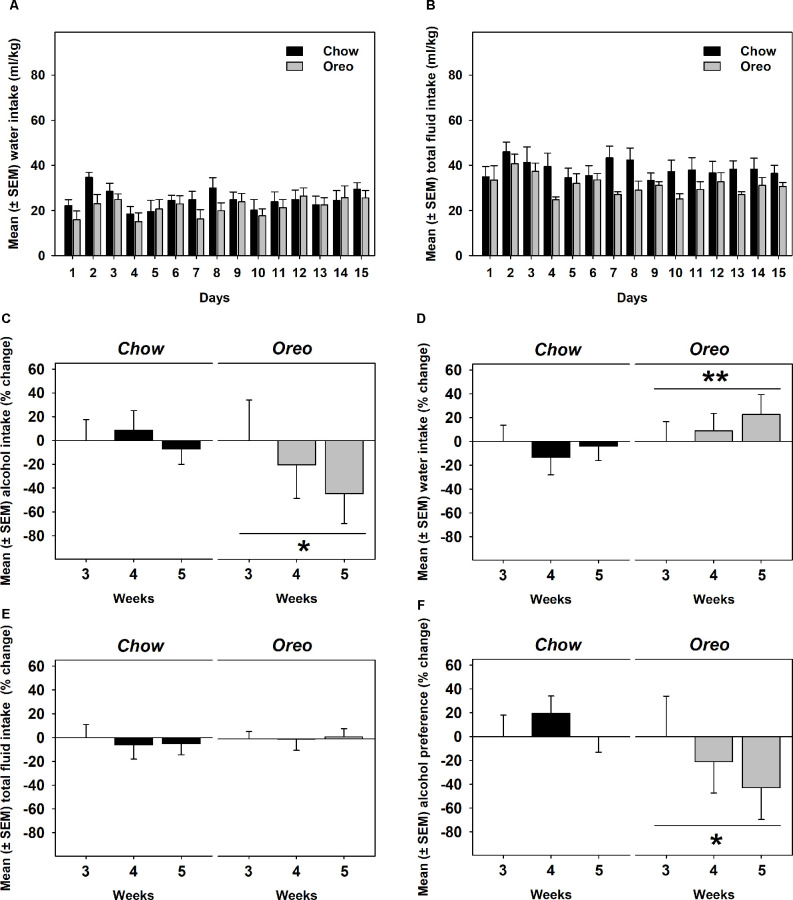
Water, total fluid intake, and alcohol preference following suspension of patterned HPF cycling. Data compares the mean (± SEM) **(A)** water and **(B)** total fluid intake (ml/kg) between chow and HPF groups and no significant between group effects were observed. As noted earlier that significantly reduced alcohol drinking behavior only emerged towards last weeks of the alcohol testing period, we evaluated average **(C)** alcohol, **(D)** water, and **(E)** total fluid intakes during the last 3 weeks along with **(F)** alcohol preference. In the Oreo group, alcohol drinking and alcohol preference was reduced, water intake was increased but the total fluid intake did not change. On the other hand, no such changes were observed in the chow group of rats. **p < 0.01, *p < 0.05 main effect of time.

A mixed-model ANOVA also evaluated water (Figure 5A) and total fluid (Figure 5B) intake data over 5 weeks of alcohol testing period and found no significant between-group differences. Since alcohol drinking in the Oreo group gradually reduced over 5 weeks and it became only significant towards the last weeks, we evaluated changes in water and total fluid intake during the last 3 weeks. A repeated measure one-way ANOVA revealed that from week 3–5, average weekly alcohol intake remained unchanged in the chow group, whereas alcohol drinking in the Oreo group of rats gradually reduced significantly (*F*_1.415,5.659_ = 6.202, *p* < 0.05; Figure 5C). While water intake in the chow group remained unchanged, it significantly (*F*_1.729,6.915_ = 14.91, *p* < 0.01) escalated in the Oreo group of rats (Figure 5D). Total fluid intake did not change under these conditions in either group (Figure 5E). These data suggest that the Oreo group of rats gradually increased their water intake during the same time when their alcohol intake was reducing and together, their fluid intake remained unchanged during the last alcohol testing weeks. These data also suggest reduced alcohol preference in the Oreo group of rats, which was supported by the repeated measure one-way ANOVA (Figure 5F) to be significant (*F*_1.369,5.477_ = 8.224, *p* < 0.05), whereas alcohol preference remained unchanged during this testing period for chow group of rats.

**Figure 6:**
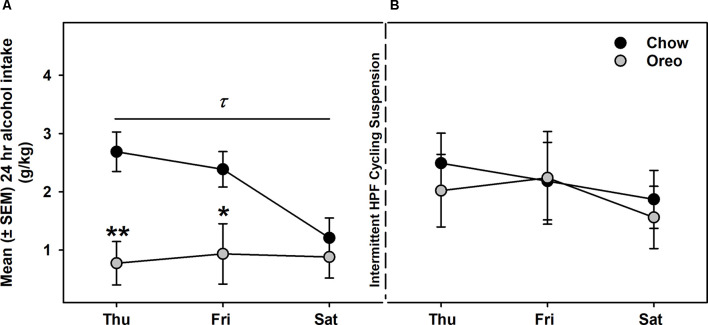
Alcohol intake following suspension of patterned HPF cycling. Data compares the mean (± SEM) alcohol intake (g/kg) between **(A)** the 5th week of regular alcohol testing and **(B)** after a week of patterned HPF cycling suspension. While the Oreo group had a significantly reduced alcohol consumption as compared to chow in the 5th week, they returned to the chow controls drinking level when the patterned HPF cycling was suspended. ^τ^*p* < 0.05 main effect of diet. *p < 0.05, **p < 0.01 compared to chow controls.

Next, we evaluated alcohol drinking following the suspension of intermittent HPF cycling. Compared to the alcohol drinking (week 5 in Figure 4) before suspension (Figure 6A), alcohol intake in the Oreo group was not significantly different and returned to the chow controls drinking level (Figure 6B).

### HPF Patterned Feeding Selectively Affected Dopamine in the NAc

**Figure 7:**
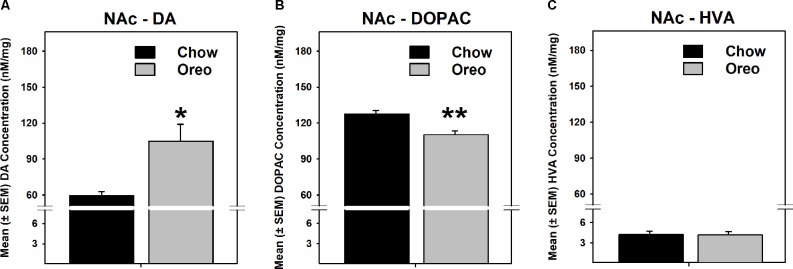
Effect of patterned feeding of HPF on dopamine levels in the NAc. Data represent mean (± SEM) concentrations (nM/mg of tissue wt) of **(A)** DA, **(B)** DOPAC and **(C)** HVA in the nucleus accumbens (NAc). DA levels were significantly elevated, whereas DOPAC levels were significantly reduced in the NAc of the Oreo group of rats compared to the chow controls. HVA levels were not significantly different between groups. *p < 0.05, **p < 0.01 compared to chow. DA, dopamine; DOPAC, 3,4-Dihydroxyphenylacetic acid.

The mesolimbic and mesocortical dopamine system is highly implicated in reward processing and affect. Given our behavioral data showing decreased ethanol intake in rats exposed to HPF patterned feeding, we quantified dopamine tissue content in regions receiving dopamine inputs from the VTA, namely, the NAc, mPFC, OFC, and amygdala. Dopamine content in the NAc (Figure 7A) was significantly greater in Oreo group of rats compared to the chow control rats (*t*_(8)_ = 3.146; *p* = 0.0137). We also evaluated DA metabolites in the NAc and found that DOPAC was significantly (*t*_(8)_ = 4.275; *p* = 0.0027) reduced in the Oreo group of rats compared to the chow controls (Figure 7B). NAc HVA content was not significantly different between groups (Figure 7C). No significant (*p* > 0.05) between-group differences in dopamine or its metabolites content were observed in other brain regions (Table 1). Since the above-mentioned regions also receive serotonin inputs from the dorsal raphe, we assessed the impact of ethanol and HPF patterned feeding on serotonin levels in these regions. Serotonin or its metabolite content was not significantly (*p* > 0.05) different in any of the brain regions examined (Table 2).

**Table 1:** Effect of patterned feeding of HPF on dopamine, DOPAC, and HVA levels in the brain reward circuitry.

Brain region	Chow	Oreo	*p*-value
**DOPAMINE**
mPFC	2.13 (± 0.29)	2.18 (± 0.06)	0.87
OFC	3.14 (± 0.10)	4.85 (± 0.96)	0.15
NAc	59.71 (± 3.06)	104.93 (± 14.04)	0.03*
Amyg	3.06 (± 0.85)	4.32 (± 1.62)	0.57
**DOPAC**
mPFC	2.94 (± 1.11)	2.33 (± 0.33)	0.62
OFC	6.09 (± 0.62)	25.19 (± 12.52)	0.20
NAc	127.64 (± 2.70)	110.32 (± 3.02)	0.003**
Amyg	27.29 (± 13.79)	5.03 (± 0.88)	0.21
**HVA**
mPFC	9.05 (± 2.01)	8.26 (± 1.52)	0.76
OFC	5.05 (± 1.16)	5.96 (± 0.99)	0.58
NAc	4.23 (± 0.48)	4.18 (± 0.47)	0.94
Amyg	14.11 (± 0.69)	20.03 (± 3.00)	0.14

Data represent mean (± SEM) dopamine and its metabolites (DOPAC and HVA) concentrations (nM/mg of tissue wt) in the medial prefrontal cortex (mPFC), orbitofrontal cortex (OFC), nucleus accumbens (NAc), and amygdala (Amyg) of Chow and Oreo group of rats. *p < 0.05, **p < 0.01 compared to chow. HPF, hyper-palatable food; DOPAC, 3,4-Dihydroxyphenylacetic acid; HVA, homovanillic acid.

**Table 2:** Effect of patterned feeding of HPF on 5-HT and 5-HIAA levels in the brain reward circuitry.

Brain region	Chow	Oreo	*p*-value
**5-HT**
mPFC	1.19 (± 0.19)	1.22 (± 0.09)	0.89
OFC	10.98 (± 0.83)	10.70 (± 0.52)	0.78
NAc	4.69 (± 0.70)	4.64 (± 0.31)	0.96
Amyg	0.88 (± 0.17)	1.04 (± 0.03)	0.44
**5-HIAA**
mPFC	60.41 (± 8.81)	59.58 (± 3.16)	0.93
OFC	64.21 (± 2.88)	61.34 (± 2.27)	0.47
NAc	102.21 (± 6.22)	89.76 (± 7.98)	0.25
Amyg	70.10 (± 9.21)	65.19 (± 4.38)	0.65

*Data represent mean (± SEM) serotonin (5-HT) and its metabolite (5-HIAA) concentrations (nM/mg of tissue wt) in the medial prefrontal cortex (mPFC), orbitofrontal cortex (OFC), nucleus accumbens (NAc), and amygdala (Amyg) of Chow and Oreo group of rats*.

## Discussion

The present study’s goal was to evaluate the impact of prolonged patterned feeding of hyper-palatable real-world food (Oreo double stuffed cookies) on alcohol drinking and alterations in the central monoamine levels. We found that HPF intermittent access induced patterned feeding and reduced alcohol drinking in rats. In addition, dopamine concentration was significantly elevated in the NAc of rats receiving patterned feeding of HPF compared to the chow controls. These data collectively suggest that patterned feeding of HPF reduces alcohol drinking, potentially by modulating dopaminergic neurotransmission in the brain reward circuitry in rats.

We and others have utilized an intermittent palatable diet access model to induce sustained bouts of caloric overconsumption and underconsumption, a hallmark of dysregulated/binge-like feeding behavior (Davis et al., 2007; Corwin and Babbs, 2012; Sirohi et al., 2017b; Villavasso et al., 2019). It is important to note that our intermittent feeding paradigm is distinct from *ad libitum* protocols that induce acute bouts of caloric overconsumption, which are not sustained and lead to an increase in body weight. In our paradigm, rats display sustained bouts of hyperphagia but no body weight gain; therefore, our results are not confounded by the presence of an obese phenotype. In the present study also, rats receiving intermittent access to Oreo cookies developed a feeding pattern of overconsumption on HPF access days (Mon, Tue, and Wed) and underconsumption on chow only access days (Thu and Fri; Figures 2A–E). It is possible that restricting access to palatable food could facilitate overconsumption of palatable food intake (Fisher and Birch, 1999); in fact, a previous study utilizing a similar approach reported an escalation in overconsumption following renewed access to a palatable diet over a 5 weeks period (Cottone et al., 2009). While the caloric intake in the present study was significantly higher on the first day of renewed HPF access compared to the second and third days, no escalation in this feeding behavior was seen over a period of 8 weeks (Figures 2A,B). We also evaluated average weekly caloric intake on Oreo access days across 8 weeks and found no evidence of escalated feeding behavior.

Preference for a high-sugar or high-fat diet under acute abstinence conditions (24 h following termination of HPF cycling) was also examined. While HFD was preferred over HSD by both chow and Oreo groups, there was no between-group difference in caloric intake (Figures 3A–D), suggesting that HPF’s patterned feeding did not induce a compulsive feeding phenotype. In the present study, rats in the experimental group had a choice between chow and Oreo cookies, which was not the case in Cottone et al. (2009) where animals in the intermittent access group had access to a palatable diet only during binge episodes and displayed compulsive feeding behavior. It is important to note that free choice palatable diet access mimics real-world situations to model neurobiological and behavioral consequences of overconsumption of palatable diets, which could differ if the palatable diet is provided as the only choice (Slomp et al., 2019).

Both animals and human studies have shown that disordered eating behavior shares many similarities and neurobiological characteristics of alcohol and substance use disorders (Cottone et al., 2009; Gearhardt et al., 2009; Volkow et al., 2012) and individuals who engage in such problematic eating behavior are at higher risk for developing alcohol use disorder, overweight/obesity, and worsening depressive symptoms (Ross and Ivis, 1999; Swanson et al., 2011; Skinner et al., 2012; Mehlig et al., 2018). Considering that hyper-palatable food is typically consumed during dysregulated eating episodes and activate similar brain reward circuitry as of drugs of abuse, including alcohol, several studies have examined the impact of overconsumption of palatable diets on alcohol (Pekkanen et al., 1978; Yung et al., 1983; Mitchell et al., 1985; Krahn and Gosnell, 1991; DiBattista and Joachim, 1999; Avena et al., 2004; Carrillo et al., 2004; Stickel et al., 2016; Takase et al., 2016; Cook et al., 2017; Gelineau et al., 2017; Sirohi et al., 2017b; Villavasso et al., 2019). While still unclear, many studies have observed a reduction in alcohol drinking (Pekkanen et al., 1978; Forsander and Sinclair, 1988; DiBattista and Joachim, 1999; Takase et al., 2016; Cook et al., 2017; Gelineau et al., 2017; Sirohi et al., 2017a,b; Constant et al., 2018; Villavasso et al., 2019; Shah et al., 2020). Paradoxical effects of HPF feeding on alcohol drinking could be attributed to palatable diets with different macronutrient compositions (high-fat or high-sugar), palatable diets exposure duration (acute, intermittent, or chronic), and alcohol testing conditions (Brutman et al., 2020). It is important to note that majority of these studies utilized commercially available purified diets high in sugar or fat. Therefore, the impact of binge-like intake of a real-world hyper-palatable food on alcohol drinking remained to be investigated and was the primary goal of the present manuscript.

In order to examine the impact of withdrawal from an extended intermittent palatable diet exposure on alcohol drinking, alcohol intake was evaluated on chow access days following 10 weeks of patterned HPF feeding. Interestingly, rats in the chow group displayed acute deprivation-induced escalated alcohol drinking (24 h) on renewed access to alcohol (Thursday), which gradually reduced to baseline levels over 3 days (Figures 4A,B). On the other hand, rats in the Oreo group were not only protected from this effect but also their 24 h alcohol intake gradually decreased, with significantly decreased alcohol drinking observed on the 4th and 5th week of alcohol testing (Figures 4A,C). Furthermore, 2 h alcohol intake (Figures 4D) was also significantly reduced in the Oreo group of rats accompanied by gradually reduced alcohol preference (Figure 5F). While we predicted that such prolonged cycling of HPF would increase alcohol drinking, surprisingly, alcohol drinking was significantly gradually reduced in the Oreo group of rats. These data are consistent with previous studies from our lab in which reduced alcohol drinking was observed following patterned feeding of a high-fat diet (Sirohi et al., 2017b; Villavasso et al., 2019). While blood alcohol levels were not assessed in the present study, a previously published study (Sirohi et al., 2017b) from our lab using Long Evans rats reported pharmacologically relevant blood alcohol levels (~25 mg/dl) following similar alcohol intake.

It has been suggested that withdrawal from fat and sugar produce fundamentally different behavioral states (Avena et al., 2009). On macronutrient levels, Oreo cookies are composed of 24% Calories from fat and 72% Calories from carbohydrates, of which 45% Calories were derived from sugar. On the other hand, high-fat diet previously used in our paradigm consisted of 40% Calories from fat and 46% Calories from carbohydrates, of which 7.9% Calories are derived from sugar (Sirohi et al., 2017a,b; Villavasso et al., 2019; Shah et al., 2020). Surprisingly reduced alcohol drinking behavior following patterned feeding to both Oreo cookies and high-fat diet suggests a role of palatability rather than macronutrient composition in reduced alcohol drinking following patterned feeding of HPF as reported by a recent study from our lab (Shah et al., 2020).

While reduced alcohol drinking was observed at least until 72 h following the suspension of the HPF, it was unclear how long this effect would last. To address this question, we suspended patterned HPF cycling and carried out alcohol testing exactly as done earlier on chow only access days (Thu, Fri, and Sat). Interestingly, alcohol drinking in the Oreo group of rats increased compared to the last alcohol drinking session and was not significantly different from chow controls (Figure 6B). These data are similar to what we had reported recently when alcohol drinking gradually increased to the level of chow controls within a week following intermittent high-fat diet suspension, suggesting that acute availability of a palatable diet is critical to observe reduced alcohol drinking behavior (Villavasso et al., 2019). In this context, an alternative explanation of reduced alcohol drinking following patterned feeding of a palatable diet could be the caloric overload before alcohol testing as a contributing factor in reduced alcohol drinking. However, previous studies from our lab have repeatedly shown that reduced alcohol drinking following patterned high-fat diet feeding is seen on days when rats did not restrict calories voluntarily (Sirohi et al., 2017b; Villavasso et al., 2019). On a similar note, alcohol drinking was gradually reduced over 5 weeks on a patterned HPF feeding cycle, whereas this behavior disappeared within a week following HPF suspension. Importantly, when tested under similar conditions, feeding peptides remained unchanged following patterned feeding of a high-fat diet (Villavasso et al., 2019). These data collectively suggest that energy homeostasis mechanisms are less likely to drive reduced alcohol drinking following patterned HPF feeding.

This notion also aligns with the data from a recent study from our lab in which selective alterations in the neurotransmitter receptors gene expression was observed in the brain reward circuity compared to the brain region involved in energy homeostasis following patterned feeding of a palatable diet (Villavasso et al., 2019). Mesocorticolimbic dopamine neuronal connectivity from VTA to NAc is highly implicated in food and drug reward processing (Wise, 2006), and palatable foods rich in fat and sugar increase extracellular dopamine in the NAc similar to drugs of abuse (Hernandez and Hoebel, 1988; Rada et al., 2005; Liang et al., 2006). While studies have reported blunted DA release following repeated/chronic palatable food/solution exposure, limited/intermittent access to sugar and fats repeatedly triggers increased DA release in the NAc (Bassareo and Di Chiara, 1997; Rada et al., 2005; Liang et al., 2006). Similarly, increased DA turnover and enhanced behavioral responses to psychostimulants have been registered following intermittent/restricted access to palatable diet (Hajnal and Norgren, 2002, but see Moore et al., 2020). However, prolonged intermittent or chronic access to palatable diets have been shown to reduce extracellular DA levels in the NAc, referred to as deficits in the mesolimbic dopamine neurotransmission (Geiger et al., 2009; Fordahl et al., 2016). While their extracellular DA levels were low, rats in the intermittent palatable diet group display increased behavioral sensitization and a greater increase in extracellular levels following the psychostimulants challenge (Fordahl et al., 2016). These effects are hallmarks of a hypodopaminergic state at baseline with an overactive dopamine system in response to external stimuli—largely due to greater dopamine availability as a result of enhanced synthesis (Mathews et al., 2009; Karkhanis et al., 2016, 2019).

Although it is unclear if extracellular levels were different, the present study found greater DA and attenuated DOPAC levels selectively in the NAc homogenates of the Oreo group compared to the chow controls rats (Figure 7A). It is possible that increased NAc DA availability in the Oreo group of rats promotes heightened sensitivity to the intoxicating effects of alcohol in the Oreo group of rats, thereby reducing the amount of alcohol needed to achieve a state similar to the chow controls, a hypothesis that needs further investigation. Since rats in the Oreo group were voluntarily restricting calories on alcohol testing days, reduced alcohol intake could reflect enhanced reward sensitivity. It is important to note that brain sections were collected on chow only access days ~24 h following patterned palatable food intake ended and >100 h following termination of alcohol drinking. While animals had no access to alcohol on that day, previous alcohol testing occurred under identical conditions on the chow only access days. Since both alcohol anticipation and ingestion increase extracellular DA levels in the NAc (Weiss et al., 1993), increased DA content in the NAc could reflect anticipation of alcohol reward. Future studies are needed to precisely understand the role of mesolimbic DA neurotransmission in regulating alcohol intake following this paradigm. Future studies are also needed to identify any sex differences in the impact of patterned feeding of HPF on alcohol drinking.

In conclusion, the present study identifies that patterned feeding of hyper-palatable food reduces alcohol drinking in rats, and alterations in the dopaminergic neurotransmission within mesolimbic circuitry could mediate reduced alcohol drinking behavior following patterned feeding. These data are consistent with previous studies from the lab and systematically replicates reduced alcohol drinking following patterned feeding a palatable high-fat diet and extend these observations to real-world food.

## Data Availability Statement

The raw data supporting the conclusions of this article will be made available by the authors, without undue reservation.

## Ethics Statement

The animal study was reviewed and approved by Institutional Animal Care and Use Committee guidelines at the Xavier University of Louisiana.

## Author Contributions

SS was responsible for conceptualization, funding acquisition, methodology, investigation, and formal analysis. SS, ZL, and KS conducted feeding and alcohol drinking experiments, wrote original draft and final version of the manuscript. LB and AK conducted HPLC analysis and wrote corresponding sections. All authors contributed to the article and approved the submitted version.

## Conflict of Interest

The authors declare that the research was conducted in the absence of any commercial or financial relationships that could be construed as a potential conflict of interest.

## Publisher’s Note

All claims expressed in this article are solely those of the authors and do not necessarily represent those of their affiliated organizations, or those of the publisher, the editors and the reviewers. Any product that may be evaluated in this article, or claim that may be made by its manufacturer, is not guaranteed or endorsed by the publisher.
